# Tumor thrombus of inferior vena cava in patients with renal cell carcinoma – clinical and oncological outcome of 50 patients after surgery

**DOI:** 10.1186/1756-0500-5-264

**Published:** 2012-06-01

**Authors:** Daniel Claudius Vergho, Andreas Loeser, Arkadius Kocot, Martin Spahn, Hubertus Riedmiller

**Affiliations:** 1 Department of Urology, Julius Maximilian University Medical School, Oberduerrbacher Str. 6, D-97080, Würzburg, Germany

**Keywords:** Renal cell carcinoma, Inferior vena cava, Thrombectomy, Tumor thrombus

## Abstract

**Background:**

To evaluate oncological and clinical outcome in patients with renal cell carcinoma (RCC) and tumor thrombus involving inferior vena cava (IVC) treated with nephrectomy and thrombectomy.

**Methods:**

We identified 50 patients with a median age of 65 years, who underwent radical surgical treatment for RCC and tumor thrombus of the IVC between 1997 and 2010. The charts were reviewed for pathological and surgical parameters, as well as complications and oncological outcome.

**Results:**

The median follow-up was 26 months. In 21 patients (42%) distant metastases were already present at the time of surgery. All patients underwent radical nephrectomy, thrombectomy and lymph node dissection through a flank (15 patients/30%), thoracoabdominal (14 patients/28%) or midline abdominal approach (21 patients/42%), depending upon surgeon preference and upon the characteristics of tumor and associated thrombus. Extracorporal circulation with cardiopulmonary bypass (CPB) was performed in 10 patients (20%) with supradiaphragmal thrombus of IVC. Cancer-specific survival for the whole cohort at 5 years was 33.1%. Survival for the patients without distant metastasis at 5 years was 50.7%, whereas survival rate in the metastatic group at 5 years was 7.4%. Median survival of patients with metastatic disease was 16.4 months.

On multivariate analysis lymph node invasion, distant metastasis and grading were independent prognostic factors. There was no statistically significant influence of level of the tumor thrombus on survival rate. Indeed, patients with supradiaphragmal tumor thrombus (n = 10) even had a better outcome (overall survival at 5 years of 58.33%) than the entire cohort.

**Conclusions:**

An aggressive surgical approach is the most effective therapeutic option in patients with RCC and any level of tumor thrombus and offers a reasonable longterm survival. Due to good clinical and oncological outcome we prefer the use of CPB with extracorporal circulation in patients with supradiaphragmal tumor thrombus. Cytoreductive surgery appears to be beneficial for patients with metastatic disease, especially when consecutive therapy is performed. Although sample size of our study cohort is limited consistent with some other studies lymph node invasion, distant metastasis and grading seem to have prognostic value.

## Background

Renal cell carcinoma (RCC) represents 3% of all solid neoplasms seen in humans [1]. In Europe, the annual incidence of RCC is approximately 2% with increased incidence of small, localized tumors. Despite recent stage migration the detection rate of advanced-stage disease has not diminished [2]. Involvement of the renal vein or/and the inferior vena cava (IVC) has been reported in 4%-10% [3,4] of patients. When it occurs without evidence of lymph node involvement or distant metastasis, surgery offers the only potential cure [5]. Meanwhile, there are several reports of larger series of patients who underwent radical surgery for RCC with inferior vena caval involvement, with reported 5-year survival rates of 34% to 72% [4,6,7]. The role of nephrectomy and thrombectomy in case of lymph node involvement or distant metastasis is not well defined [4,6]. In symptomatic patients (intractable edema, cardiac dysfunction, abdominal pain, hematuria) removal of tumor thrombus may provide better quality of life, even if it does not cure the patient [1]. Combination of cytoreductive surgery and targeted therapy may prolong survival [8,9]. The potential value of using multitargeted receptor tyrosine kinase inhibitors in adjuvant or even neoadjuvant setting is unclear.

Prognostic significance of the cephalad extension of the tumor thrombus has been discussed extensively and controversially in the literature, and it is difficult to compare various series because of differences in selection of patients and related covariables [10]. Although some series have indicated it may be a negative prognostic factor [3,11], other authors report no difference in survival of patients with supradiaphragmatic versus infradiaphragmatic tumor thrombi as long as the tumor is otherwise confined [12].

The present study reports our experience of surgical treatment of patients with RCC and venous thrombus of the IVC, with a particular focus on clinical and oncological outcomes.

## Materials and methods

From April 1997 to March 2010 50 patients, 36 men and 14 women, with a mean age of 65 years (47 to 85 y.) underwent resection of a RCC with extension of tumor thrombus into the IVC (Stage T3b/c according to UICC 2002). The charts of our patients were reviewed retrospectively for demographics, clinical presentation, preoperative staging and laboratory values (hemoglobin, thrombocyte count, lactate dehydrogenase (LDH), C reactive protein (CRP)), pathology as well as surgical parameters (operation time, number of blood transfusions, complications, hospitalisation time). Long-term follow-up data were collected during check-up visits and additional telephone interviews with the urologist of the patient. Because patients were treated according to the guidelines and current state of art a statement of ethical approval is not required.

Preoperatively, all patients underwent routine blood tests, ultrasound, chest and abdominal computed tomography (CT) and/or abdominal magnetic resonance imaging (MRI) and/or bone scintigraphy. Clinical and pathological staging was performed using the TNM classification (2002 TNM classification of malignant tumors (UICC), 6^th^ edition). Tumor grade was classified according to the Fuhrman grading system [13].

**Figure 1:**
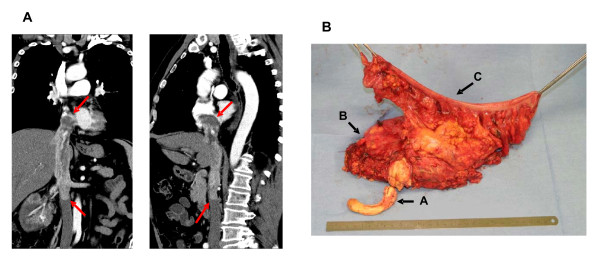
**Examples for advanced tumor thrombi. A** Preoperative CT-scan of a 65 year old male with a Level IV tumor thrombus extending to the right ventricel. This patient lives 27 months after surgery without evidence of disease; **B** Surgical specimen including “en-block” resection of Level IV tumor thrombus (A), kidney with tumor (B) and left hemicolon (C). This patient, presented with pulmonal metastasis at time of surgery, lives 43 months after surgery receiving multikinase inhibitors.

The level of tumor thrombus was classified according to the Mayo classification [14] (Table 1). 5 patients (10%) had a level I tumor thrombus in the IVC, 16 (32%) a level II thrombus, 19 (38%) a level III thrombus, and 10 (20%) a level IV thrombus (Figure 1A).

**Table 1:** The Mayo classification of macroscopic venous invasion in renal cell carcinoma

**Level I**	Tumor thrombus is either at the entry of renal vein or within the IVC < 2 cm from the confluence of renal vein and IVC.
**Level II**	Tumor thrombus extends within the IVC > 2 cm above the confluence of renal vein and IVC, but still remains below the hepatic veins.
**Level III**	Tumor Thrombus involves the intrahepatic IVC.
**Level IV**	Tumor thrombus extends above diaphragm or into the right atrium.

### Surgical and therapeutic approach

All patients underwent radical nephrectomy, thrombectomy and lymph node dissection through a flank (15 patients/30%), thoracoabdominal (14 patients/28%) or midline abdominal approach (21 patients/42%), depending upon surgeon preference and upon the characteristics of tumor and associated thrombus.

11 patients (22%) were operated in cooperation with the Department of Cardiothoracic Surgery of the University Hospital Wuerzburg. To access the right atrium in these patients sternotomy was performed. Extracorporal circulation with cardiopulmonary bypass (CPB) was performed in 10 patients (20%) with supradiaphragmal thrombus of IVC. In one patient with Level III thrombus CPB was planned but intraoperatively assumed as not necessary.

Partial resection and replacement of IVC was necessary in 5 cases. One patient additionally to radical nephrectomy and thrombectomy required resection of the left hemicolon because of tumorinfiltration (Figure 1B).

Postoperatively, 17 patients (34%) received cytokine treatment and 3 (6%) were treated by multitargeted therapy.

One patient was treated neoadjuvantly with tyrosin kinase inhibitor.

### Statistic analysis

Actuarial patient survival and disease-free survival were estimated using Kaplan-Meier method. Subgroup differences were tested by two sided log-rank tests. The prognostic significance of certain factors was assessed by the Cox proportional hazards regression model. In all tests p < 0.05 was considered to indicate statistical significance. Statistical calculations were carried out using SPSS (version15.0).

## Results

38 tumors originated from the right kidney and 12 from the left. Median tumor size was 10.1 cm (range 5 to 17 cm). Involvement of the IVC wall (histologically proven infiltration) was discovered in 14 patients (28%). In 21 patients (42%) distant metastases were already present at time of surgery. The lung was the most prevalent location of metastases in 14 (28%) cases, in two cases combined with other locations (bone and liver). 5 patients (10%) had extrapulmonary metastasis (1 liver, 1 adrenal gland, 1 mediastinal lymphnodes, 2 brain). The tumor was symptomatic in 35 patients (70%), whereas 15 (30%) had incidental findings. Most common observed presenting symptoms were gross hematuria (28%), flank or abdominal pain (22%) and systemic signs (18%). Three patients presented with edema of lower limbs and one patient with pulmonary embolism. One patient (67-year-old male) presented with solid mass (3.5 cm) in his left kidney and tumor thrombus Level III with caudal extension into both iliacal veins, on the left side even into the femoral vein. Due to extension of tumor thrombus and comorbidity (chronic renal failure, COPD, diabetes mellitus, hypertension, obesity) he was assessed as inoperable and treated with tyrosine kinase inhibitors for 5 weeks (sorafenib 400 mg twice daily). Restaging showed no change of the solid mass of left kidney, but complete reduction of tumor thrombus and obliteration of IVC. Thereafter surgical resection was performed successfully. Table 2A lists the characteristics of the study population.

**Table 2:** **Patient characteristics and results (n = 50). A Summary of clinical and histopathological data. B Complications (grade according to the Clavien classification of surgical complications [**[15]**]). C Variables used on univarate analysis. D Prognostic factors in multivariate analysis (Cox proportional hazards regression model).**

**A Summary of clinical and histopathological data**				
**Characteristics**	**Entire Group (n = 50)**	**Pat. without Metastases (n = 29/58%)**	**Pat. with Metastases (n = 21/42%)**	**p-value for difference**
Sex				
Female	14 (28%)	12 (41%)	2 (9.5%)	p = 0.0094
Male	36 (72%)	17 (59%)	19 (90%)	
Laterality				
Left	12 (24%)	4 (14%)	8 (38%)	p = 0.047
Right	38 (76%)	25 (87%)	13 (62%)	
Symptomatic	35 (70%)	18 (62%)	17 (81%)	
Gross hematuria	14 (28%)	9 (31%)	5 (24%)	
Flank or abdominal pain	11 (22%)	5 (17%)	6 (29%)	
Edema lower limbs	3 (6%)	1 (3,5%)	2 (9.5%)	
Pulmonary embolism	1 (2%)	0 (0%)	1 (4.7%)	p = 0.84
Systemic symptoms (lost of weight, night sweat, anemia, etc.)	9 (18%)	4 (14%)	5 (24%)	
Tumor Grade				
G1	0 (0%)	0 (0%)	0 (0%)	
G2	32 (64%)	22 (76%)	10 (48%)	p = 0.04
G3	18 (36%)	7 (24%)	11 (52%)	
G4	0 (0%)	0 (0%)	0 (0%)	
Histological Type				
Clear cell	39 (78%)	22 (76%)	17 (59%)	
Papillary	7 (14%)	4 (14%)	3 (14%)	p = 0.95
Sarcomatoid	2 (4%)	1 (3,5%)	1 (4,7%)	
Others	2 (4%)	2 (7%)	0 (0%)	
T stage				
T3b	39 (78%)	21 (72%)	18 (86%)	p = 0.12
T3c	10 (20%)	8 (28%)	2 (9.5%)	
T4	1 (2%)	0 (0%)	0 (0%)	
N stage				
N0	38 (76%)	23 (79%)	15 (71%)	
N1	4 (8%)	4 (14%)	0 (0%)	p = 0.018
N2	8 (16%)	2 (7%)	6 (29%)	
Level of Tumor Thrombus				
Level I	5 (10%)	3 (10%)	2 (9.5%)	
Level II	16 (32%)	11 (38%)	5 (24%)	p = 0.093
Level III	19 (38%)	7 (24%	12 (57%)	
Level IV	10 (20%)	8 (28%)	2 (9.5%)	
Infiltration of IVC				
yes	14 (28%)	10 (35%)	4 (19%)	p = 0.22
no	36 (72%)	19 (65%)	17 (81%)	
Infiltration of perinephritic tissue				
yes	29 (58%)	12 (41%)	17 (81%)	p = 0.013
no	21 (42%)	17 (59%)	4 (19%)	
**B** Complications (grade according to the Clavien classification of surgical complications [15])				
**Complications**	**Grade**	**n**	**%**	**Management**
Acute retroperitoneal hemorrhage	IV	3	6%	Relaparotomy
Pericardial Effusion	II	1	2%	Conservatively
Postop. Hematoma/wound infection	II	2	4%	Conservatively
Ileus	II	1	2%	Conservatively
**Total**		**7**	**14%**	
**C Variables used on univarate analysis**				
**Prognostic factor**			**p-value (univariate analysis)**	
pT			p = 0.99	
pN			p = 0.00007	
M			p = 0.026	
R			p = 0.19	
Histology			p = 0.11	
Grading			p = 0.042	
Infiltration perirenal tissue			p = 0.035	
Infiltration wall vena cava			p = 0.012	
Age			p = 0.28	
Size of tumor			p = 0.019	
Level of tumor thrombus (Mayo)			p = 0.31	
Extracorporal circulation			p = 0.4	
Hemoglobin			p = 0.3	
Thrombocytes			p = 0.18	
Ca			p = 0.9	
LDH			p = 0.48	
CRP			p = 0.014	
Alcalic phosphatase			p = 0.076	
Age			p = 0.28	
Adjuvant Treatment			p = 0.15	
**D Prognostic factors in multivariate analysis (Cox proportional hazards regression model)**				
**Prognostic factor**			**p-value (multivariate analysis)**	
Grading			p = 0.021	
Distant Metastasis			p = 0.00091	
Lymph node invasion			p = 0.000003	

Mean operative time was 4.4 hours (2.25 – 10 h). 38 patients received blood transfusion, with a mean of 4.5 units (0 to 13). The mean hospital stay was 19 days (11 to 33). There was no intra- or perioperative (30-day postoperative period) death. In three patients relaparatomy was performed because of acute retroperitoneal hemorrhage. In addition two patients were reported with postoperative hematoma with wound infection, one patient with pericardial effusion and one patient with ileus. Conservative management was possible in all of these 4 patients. Beside this according to the Clavien classification [15] no complications, especially no pulmonary embolism, have been observed. Depending on the approach we observed 5 complications in patients who underwent sternotomy and abdominal midline incision, including 2 relaparotomies, 1 hematoma, 1 pericardial effusion, 1 ileus. 1 relaparatomy was necessary after an abdominal midline incision and 1 hematoma, respectively wich was seen after a flank incision. Complications are presented in Table 2B.

Histopathological examination revealed RCC of clear cell variety in 39 patients (78%), papillary in 7 (14%), sarcomatoid in 2 and mixed in 2. There were lymphnode metastases in 12 patients (24%).

**Figure 2:**
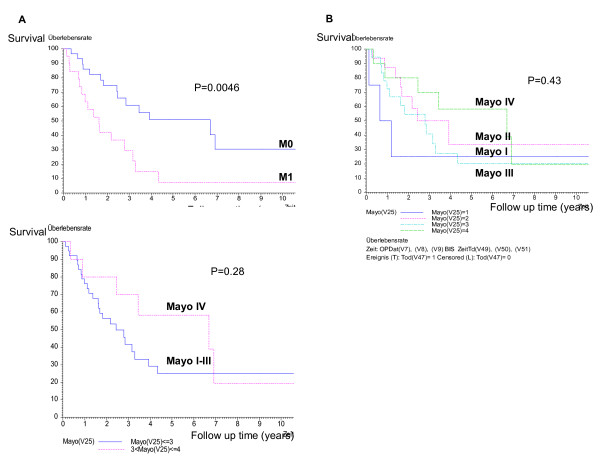
**The cancer specific rates in patients with RCC and tumor thrombus of IVC. A** Kaplan-Meier cancer specific classified by tumor extension (M0 subgroup vs M1 subgroup, p = 0.046); **B** Kaplan-Meier cancer specific survival classified by level of the tumor thrombus (p = 0,43); **C** Kaplan-Meier cancer specific survival classified by level of the tumor thrombus (Mayo I-III subgroup vs Mayo IV subgroup, p = 0,28).

The median follow-up was 26 months (1 to 127 months). 30 deaths have been observed so far, 29 of them due to tumor progression. Cancer-specific survival for the whole cohort at 1, 2 and 5 years was 76.9%, 61.6% and 33.1%, respectively. Survival for the patients without distant metastasis at time of surgery was 85.9%, 74.7% and 50.7% (median survival 31 months), whereas survival rates in the metastatic group were 63.3%, 42.2% and 7.4%, as shown in Figure 2A. Median survival of this group was 16.4 months (493 days).

Univariate analysis showed that lymph node invasion (p = 0,00007), distant metastasis (p = 0.026), grading (p = 0,042), infiltration of perirenal tissue (p = 0,035), infiltration of vena cava wall (p = 0,012), tumor size (p = 0,019) and CRP (p = 0,014) were statistically significant predictors for survival (Table 2C). On multivariate Cox regression analyses grading (p = 0.021), distant metastasis (p = 0.00091) and lymph node invasion (p = 0.000003) remained statistically independent prognostic factors, as summarized in Table 2D.

There was no statistically significant influence of level of the tumor thrombus on survival rate (Figure 2B and 2C). Indeed, the subgroup of patients with supradiaphragmal tumor thrombus (Level IV) (n = 10) even had a better outcome with an estimated survival rate of 58.33% at 5 years. The median follow-up of this subgroup was 59.5 months (4 to 127 months). 4 of the 10 patients are still alive, 3 of them without evidence of disease (32–127 months after surgery, average 79 months). One patient with a solitary histologically proven metastasis of the liver currently is alive 10.6 years after nephrectomy, thrombectomy and metastasectomy without any sign of disease. However, only 2 of these 10 patients had distant metastases at time of diagnosis compared to 47.5% (19 of 40 patients) in the group of patients with infradiaphragmal tumor thrombus and primary metastatic disease.

The patient, mentioned above who was given neoadjuvant therapy with tyrosin kinase inhibitor (Sorafenib) featured pathologically a 5.5 cm clear cell RCC with signs of tumor regression, Fuhrman grade 2. Surprisingly, renal vein appeared recanalised without tumor thrombus (no histological evidence of malignancy).

## Discussion

In patients with locally advanced RCC an aggressive surgical approach is the only hope for curing. RCC extending to IVC presents a challenging surgical management problem. In patients with non-metastatic RCC and IVC involvement the 5-year survival rates range between 34% and 72% [4,6,7]. Cancio et al. reported a 5-year disease-free survival rate of 54.5% for N0M0 disease [16]. In our series the 5-year cancer-specific survival rate for patients without metastases is 50.7%, which is comparable to other studies.

Approximately one third of patients with RCC and associated tumor thrombus also show distant metastases at time of presentation. In our patient-cohort 42% of the patients with thrombus in the IVC presented with simultaneous metastases, a percentage which is higher than in other series. Cianco et al. reported median time to death of 8 months and a longest survival of 27 months in patients with metastatic disease [16]. Staehler and Brkovic reported median survival of 13 months and 2-year survival rate of 26% for this group [6]. In our analysis, 17 of 21 patients with metastatic disease died, whereas 4 patients (follow-up 1, 32, 32, 127 months) still are alive. 2-year survival rate was 42.2%, median time to death 16.4 months with a range of 1.5 to 52 months.

Several authors do not advise radical surgery for patients with metastatic disease and thrombus of the IVC, believing that the limited chance of longer survival did not justify the morbidity of such an extensive operation [17,18]. In a study of Lambert et al. patients with metastatic disease did not experience any added morbidity or mortality compared to patients without metastatic disease [19]. In two randomized trials, on the other hand, cytoreductive nephrectomy has demonstrated improved survival for patients with metastatic RCC treated additionally with interferon [20,21]. Although a survival advantage for cytoreductive nephrectomy in combination with targeted therapies has not yet been confirmed in the setting of clinical trial, the multimodal strategy has also been extrapolated to the era of targeted therapy [8,9,22]. Also, the impact of targeted therapies in a neoadjuvant setting is not defined. Especially patients with a primary unresectable tumor thrombus could possibly benefit from neoadjuvant targeted therapies. Lately, some cases of tumor and thrombus regression followed by secondary operability after neoadjuvant therapy with tyrosinkinase inhibitors have been reported [23]. In our cohort 1 patient with an initially unresectable tumor thrombus Level III was treated successfully by neoadjuvant administration of tyrosin kinase inhibitor (Sorafenib). These observations might be a signal to re-evaluate the paradigm for the management of advanced RCC especially with venous involvement. The integration of targeted therapy in a neoadjuvant setting for downstaging is an very interesting objective, considering the potential for decreased perioperative morbidity [22]. However, the benefit of neoadjuvant therapy can only be studied further within a multicenter clinical trial setting.

Several studies are concerned with prognostic factors of patients with RCC and associated thrombus in IVC. In a number of studies lymph node invasion and distant metastases were described as independent prognostic factors and their presence is known to reduce survival not only in patients with venous involvement [3,10,12,16,17]. Leibovich et al. identified the presence of perinephric fat invasion in patients with tumor thrombus as an independent prognostic factor [10]. In a recent multicenter study Wagner et. al reviewed data of 1192 patients, who underwent radical nephrectomy for T3b and T3c RCCs and reported besides IVC invasion, fat invasion, lymphnode and distant metastasis on tumor size as an additional independent prognostic factor [24]. Certainly, our study sample is limited and conclusions of independent prognostic factors can only be drawn carefully. But consistent with other studies [3,6,24], on multivariate analyses lymph node invasion, distant metastases and tumor grade seemed to be of prognostic relevance.

One of the most controversial topics in treatment of IVC tumor thrombus in RCC has been the level of thrombus and its prognostic impact on survival. The most recent TNM classification (2010) distinguishes between T3b tumors that extend into the IVC or its wall below the diaphragm and T3c tumors that extend into the IVC or its wall above the diaphragm. Some studies showed a negative impact on survival in patients with tumor thrombus involving the IVC, in particular in those with a higher cephaled extension [3,11,25]. Other reports suggest that tumor thrombus extension into the IVC is not necessarily associated with a worse prognosis [12,24,26]. The presence of IVC invasion, not the level of tumor thrombus, was identified as an independent prognostic factor in some series [24,27]. Wagner et al. [24] reported on statistically different overall survival for patients with tumor thrombus in the renal vein compared to those with IVC involvement, whereas the level of tumor thrombus in the IVC did not significantly affect the overall survival. In our series there was no significant influence of the level of tumor thrombus on survival. In fact, the subgroup of patients with a level IV tumor thrombus (n = 10), who required surgery with cardiopulmonary bypass, had a better outcome. This certainly is due to patient selection, considering that only 2 of the 10 patients had distant metastases at time of diagnosis compared to 47.5% (19 of 40 patients) with metastatic disease in the group of patients with infradiaphragmal tumor thrombus. Furthermore the subgroup of patients with supradiaphragmal tumor thrombus even had a better outcome than patients without distant metastases (n = 29) with a survival rate at 5 years of 50,7%, which underlines the safeness of the surgical approach including the use of CPB for this group of patients.

Surgical management of patients with supradiaphragmal tumor thrombus of the IVC has always been a technically challenging operation for urologists. According to literature, nephrectomy with vena caval thrombectomy is associated with a perioperative mortality rate of 3-16% [16,24,28]. Preoperative accurate imaging is mandatory to guarantee optimal surgical strategy. While clamping the infrahepatic IVC is usually well-tolerated, occlusion of the suprahepatic IVC frequently causes severe hypotension due to the decrease in venous return. The use of CPB with or without deep hypothermic circulatory arrest has been used commonly as a standard procedure [29,30]. Alternatively, the technique of venovenous bypass described for orthotopic liver transplantation has also been investigated for tumor thrombectomy during radical nephrectomy with good results [31]. The group from University of Miami described their success in managing IVC thrombi in a special technique of liver mobilisation and “milking” of the tumor thrombus toward the cavotomy, while maintaining organ perfusion [5]. Due to the low morbidity and mortality rate of patients with supradiaphragmal tumor thrombus in our cohort (no perioperative death and only 2 relaparotomies) we still favour cardiopulmonary bypass for tumor thrombi above the hepatic veins. We did not observe any other severe complications associated with CPB, including perioperative coagulopathy, hepatic failure, neurologic dysfunction or postoperative sepsis.

The present study is a retrospective review of a single institutional experience and, as such, it is limited by inherent biases. So, complication data might be limited by the information available in the patients` charts and value of multivariate analysis is limited by sample size.

## Conclusion

In the present study we show, as others have before, that an aggressive surgical approach is the most effective therapeutic option in patients with RCC at any level of tumor thrombus and offers reasonable long-term survival. Due to low morbidity and mortality rate and the good oncological outcome we prefer the use of CPB in patients with supradiaphragmal tumor thrombus.

Cytoreductive surgery may even be beneficial for patients with metastatic disease, especially when consecutive therapy - such as multitargeted approaches – is performed. Consistent with other studies, on multivariate analyses lymph node invasion, distant metastasis and tumor grade seemed to be of prognostic relevance.

## Competing interests

The authors declare that they have no competing interests.

## Authors’ Contributions

DV conceived this retrospective study and contributed in the conception, Data acquisition and statistical analysis as well as drafting the manuscript. AL performed the statistical analysis and helped to draft the manuscript. AK participated in Data acquisition, analysis and interpretation as well as development of manuscript. MS participated designing the study concept and contributed critical revision of the manuscript for scientific and factual content. HR supervised the study and participated in its conception as well as critical revision. All authors read and approved the final manuscript.
